# Crystal structure of 1-[2-(4-nitro­phen­yl)-4,5-diphenyl-1*H*-imidazol-1-yl]propan-2-ol

**DOI:** 10.1107/S2056989017011057

**Published:** 2017-08-30

**Authors:** Jim Simpson, Shaaban K. Mohamed, Adel A. Marzouk, Antar A. Abdelhamid, Mustafa R. Albayati

**Affiliations:** a Department of Chemistry, University of Otago, PO Box 56, Dunedin, New Zealand; b Faculty of Science and Engineering, Health Care Division, Manchester Metropolitan University, Manchester M1 5GD, England; c Chemistry Department, Faculty of Science, Minia University, 61519 El-Minia, Egypt; d Pharmaceutical Chemistry Department, Faculty of Pharmacy, Al Azhar University, 71515 Assiut, Egypt; e Chemistry Department, Faculty of Science, Sohag University, Sohag, Egypt; f Kirkuk University, College of Education, Department of Chemistry, Kirkuk, Iraq

**Keywords:** crystal structure, multi-substituted imidazole, hydrogen bonding, C—H⋯π inter­actions

## Abstract

The title compound, C_24_H_21_N_3_O_3_, crystallizes with two unique but closely r.m.s. overlay fit = 0.215 Å) comparable mol­ecules (1 and 2) in the asymmetric unit of the triclinic unit cell. In molecule 1, the dihedral angles between the central imidazlole ring and the benzene-ring substituents are 42.51 (9), 45.41 (9) and 56.92 (8)°, respectively. Comparable data for molecule 2 are 39.36 (10), 34.45 (11) and 60.34 (8)°, respectively. The rings at the 2-positions carry *p*-nitro substituents that subtend dihedral angles of 12.9 (4)° in mol­ecule 1 and 11.7 (4)° in mol­ecule 2 to their respective benzene ring planes. The imidazole rings also have propan-2-ol substituents on the 1-N atoms, which adopt extended conformations for the N—C—C—C chains. In the crystal, classical O—H⋯N hydrogen bonds combine with C—H⋯O, C—H⋯N and C—H⋯π(ring) hydrogen bonds and stack the molecules along the *a*-axis direction.

## Abstract 2

The mol­ecular and crystal structure of the title imidazole derivative is reported. The structure features an extensive O—H⋯N, C—H⋯O/N and C—H⋯π(ring) hydrogen-bonding network.

## Chemical context   

Imidazole compounds form the core of the structures of some well-known components of human organisms including the amino acid histidine, vitamin-B12, a component of the DNA base structure and the purines, histamine and biotin. It is also present in the structure of many natural or synthetic drug mol­ecules, for example cimetidine, azomycin and metronidazole (Kleeman *et al.*, 1999 ▸). Imidazole derivatives display an extensive range of biological activities and are thus used as anti­bacterial (Vijesh *et al.*, 2011 ▸; Lu, *et al.*, 2012 ▸), anti­cancer (Yang *et al.*, 2012 ▸; Alkahtani *et al.*, 2012 ▸), anti-tubercular (Lu, *et al.*, 2012 ▸; Lee *et al.*, 2011 ▸), analgesic (Kankala *et al.*, 2013 ▸; Gaba *et al.*, 2010 ▸) and anti-HIV agents (Zhan *et al.*, 2009 ▸). As part of an ongoing study of the synthesis of imidazole-based amino aliphatic alcohols, *e.g.* amino ethanol and amino isopropanol (Akkurt *et al.*, 2015 ▸; Mohamed *et al.*, 2013*a* ▸,*b* ▸; Jasinski *et al.*, 2015 ▸), we report here the synthesis and crystal structure of the title compound.

## Structural commentary   

**Figure 1:**
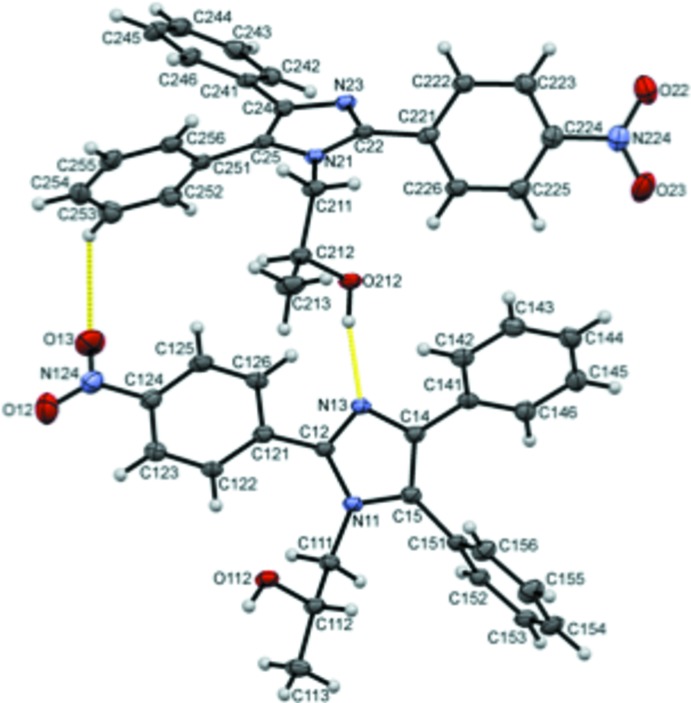
The asymmetric unit of (I), with displacement ellipsoids drawn at the 50% probability level. Hydrogen bonds between the two unique molecules are shown as yellow dashed lines.

The title compound, (I), crystallizes with two unique mol­ecules, 1 and 2, in the asymmetric unit. In the numbering scheme these mol­ecules are differentiated by leading 1 and 2 digits, respectively, Fig. 1 ▸.

**Figure 2:**
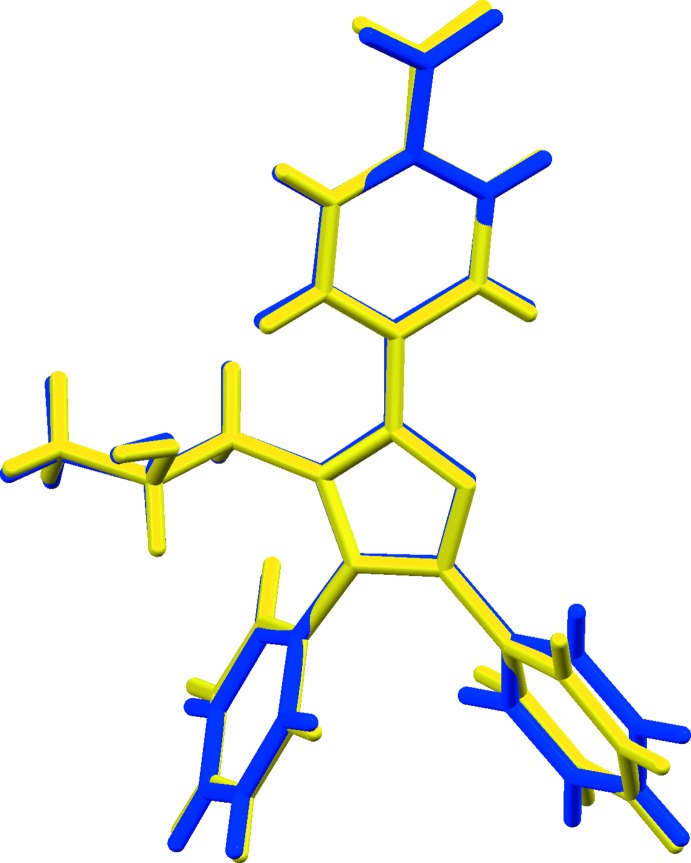
An overlay (Macrae *et al.*, 2008 ▸) of the two mol­ecules. Mol­ecule 1 is drawn in yellow with mol­ecule 2 in blue.

 The unique mol­ecules form dimers in the asymmetric unit through O212—H210⋯N13 and C253—H253⋯O13 hydrogen bonds that enclose 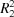
(18) rings, Fig. 1 ▸. The two mol­ecules are closely similar and an overlay, Fig. 2 ▸ (Macrae *et al.*, 2008 ▸), shows an r.m.s. deviation of 0.215 Å with relatively minor variations of the inclinations of the various substituents to the central imidazole rings.
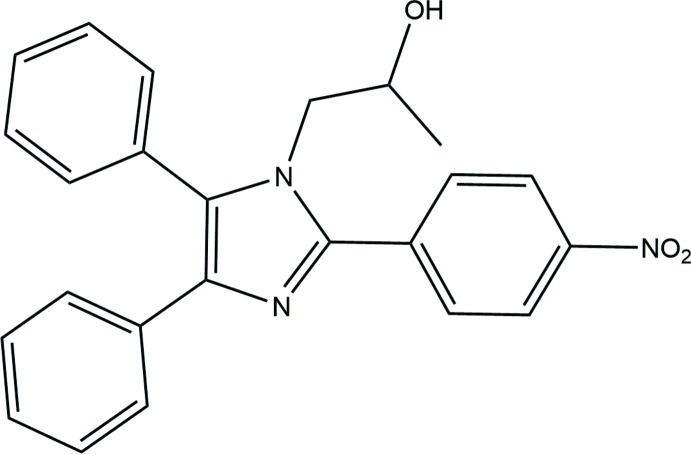


The structure consists of a basic lophine, 2,4,5-triphenyl-1*H*-imidazole, skeleton (Yanover & Kaftory, 2009 ▸), with iso­propanol substituents on the N11 and N21 atoms of the central imidazole rings. The N–C_3_chains of these substituents are planar, with an N1—C11—C12—C13 torsion angle of 173.09 (19)° in molecule 1 and 171.0 (2)° in molecule 2; these planes are inclined to the imidazole ring planes by 74.96 (12) and 74.78 (12)°. respectively. The benzene rings are inclined to the imidazole ring plane at angles of 42.51 (9) and 39.36 (10)° for C121–C126 and C221–C226, 45.41 (9) and 34.45 (11)° for C141–C146 and C241–C246 and 56.92 (8) and 60.34 (8) for C151–C156 and C251–C256, values that further attest to the close similarities between the structures of the two unique mol­ecules. Bond lengths and angles in the two mol­ecules are also similar and compare well with those found in comparable mol­ecules with iso­propanol substituents at the 1-position (Jasinski *et al.*, 2015 ▸; Mohamed *et al.*, 2017 ▸; Akkurt *et al.*, 2015 ▸).

## Supra­molecular features   

**Figure 3:**
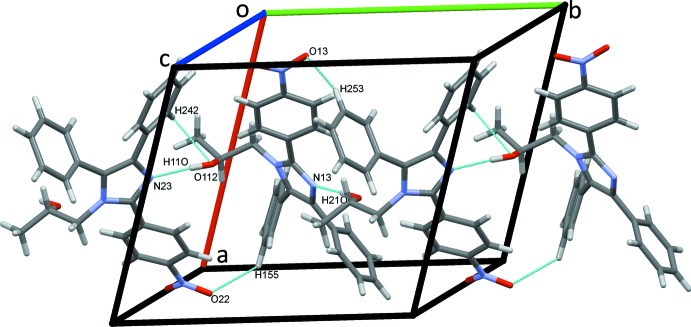
Zigzag chains of mol­ecules of (I) along *b*. In this and subsequent Figures, hydrogen bonds are drawn as dashed lines.

**Figure 4:**
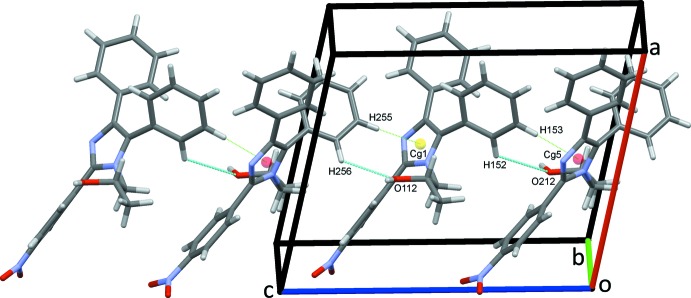
Chains of mol­ecules of (I) along *c*. C—H⋯π(ring) contacts are drawn as dotted green lines with ring centroids shown as coloured spheres.

**Figure 5:**
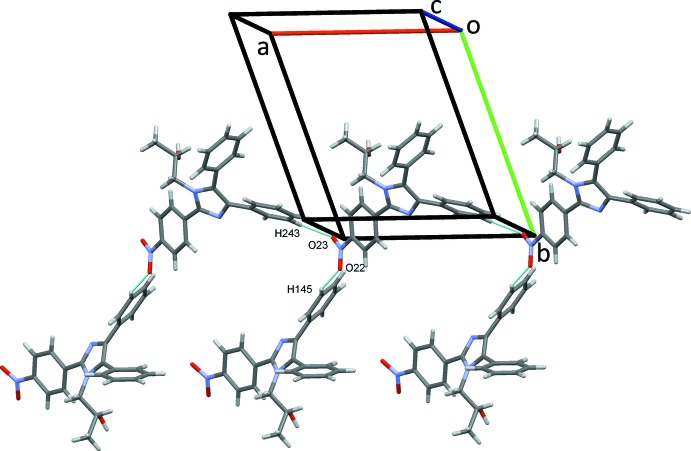
Chains of type 2 mol­ecules of (I) along *a* linked to type 2 mol­ecules, forming sheets in the *ac* plane.

**Figure 6:**
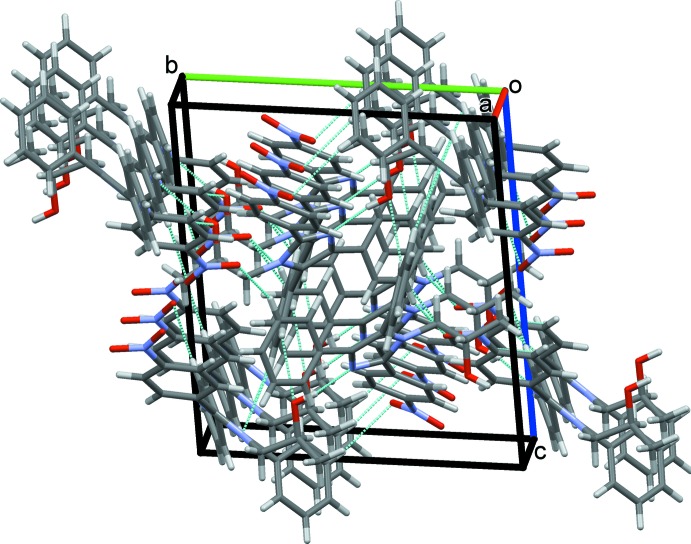
Overall packing of (I) viewed along the *a*-axis direction.

In the crystal, classical O112—H11*O*⋯N23 and O212—H21*O*⋯N13 hydrogen bonds, Table 1 ▸, bolstered by weaker C155—H155⋯O22, C242—H242⋯O112 and C253—H253⋯O13 hydrogen bonds link type 1 and type 2 mol­ecules alternately in a head-to-tail fashion into *C*(8) chains along the *b-*axis direction, Fig. 3 ▸. Chains of alternate mol­ecules also form along *c*, in this case head-to-head, due to C153—H153⋯*Cg*5 and C255—H255⋯*Cg*1 contacts (*Cg*1 and *Cg*5 are the centroids of the N11/C12/N13/C14/C15 and N21/C22/N23/C24/C25 rings, respectively) combined with C152—H152⋯O212, C153—H153⋯N21 and C256—H256⋯O112 hydrogen bonds, Fig. 4 ▸. Chains exclusively of type 2 mol­ecules form along the third axial direction *via* C243—H243⋯O23 hydrogen bonds, forming *C*(13) chains along *a*, Fig. 5 ▸. C145—H145⋯O22 hydrogen bonds link type 1 mol­ecules to these chains, stacking the mol­ecules along *a*. Overall, these numerous contacts generate layers of molecules of (I) stacked along the *a-*axis direction, Fig. 6 ▸.

## Database survey   

The Cambridge Structural Database (Version 5.38 with three updates; Groom *et al.*, 2016 ▸) shows that mol­ecules with the lophine skeleton and a CH_2_ substituent on N1 are reasonably common with 43 entries. However, restricting the search to compounds with iso­propanol substituents on N1 reduces the hits to three reports of our work to produce compounds with 4-benzoic acid (Jasinski *et al.*, 2015 ▸) and 4-chloro- (Mohamed *et al.*, 2017 ▸) and 2,5-di­chloro-substituents (Akkurt *et al.*, 2015 ▸) at the 2-position of the imidazole ring. A more recent paper, detailing the use of ionic liquids as catalysts for the preparations of similar compounds, also reports analogues with an unsubstituted phenyl ring and a 2,5-dimeth­oxy substituted benzene ring at the 2-positions (Marzouk *et al.* 2017 ▸). Other closely related derivatives have ethanol (Mohamed *et al.*, 2013*a* ▸) and *n*-propanol substituents on the N1 atom (Mohamed *et al.*, 2015 ▸).

## Synthesis and crystallization   

The title compound was prepared according to our previously reported method (Marzouk *et al.*, 2017 ▸). Crystals suitable for X-ray analysis were obtained by the slow evaporation method using ethanol as a solvent. M.p. 451–453 K, yield, 87%.

## Refinement   

Crystal data, data collection and structure refinement details are summarized in Table 2 ▸. The hydrogen atoms of the OH groups on O112 and O212 were located in a difference-Fourier map and their coordinates refined with *U*_iso_ = 1.5*U*_eq_ (O). All other atoms were refined using a riding model with *d*(C—H) = 0.95 Å for aromatic, 1.00 Å for methine and 0.98 Å for CH_2_ atoms, all with *U*_iso_(H) = 1.2*U*_eq_(C). For methyl H atoms *d*(C—H) = 0.98 Å and *U*_iso_(H) = 1.5*U*_eq_(C). One low angle reflection with *F*_o_ << *F*_c_ that may have been affected by the beamstop was omitted from the final refinement cycles.

## Supplementary Material

Crystal structure: contains datablock(s) I, global. DOI: 10.1107/S2056989017011057/hg5491sup1.cif

Structure factors: contains datablock(s) I. DOI: 10.1107/S2056989017011057/hg5491Isup2.hkl

Supporting information file. DOI: 10.1107/S2056989017011057/hg5491Isup3.cml

CCDC reference: 1565059

Additional supporting information:  crystallographic information; 3D view; checkCIF report

## Figures and Tables

**Table 1:** Hydrogen-bond geometry (Å, °) *Cg*1 and *Cg*5 are the centroids of the N11/C12/N13/C14/C15 and N21/C22/N23/C24/C25 rings, respectively.

*D*—H⋯*A*	*D*—H	H⋯*A*	*D*⋯*A*	*D*—H⋯*A*
O212—H21*O*⋯N13	0.89 (4)	1.90 (4)	2.773 (2)	168 (3)
C253—H253⋯O13	0.95	2.69	3.491 (4)	143
O112—H11*O*⋯N23^i^	0.88 (4)	1.94 (4)	2.798 (2)	165 (3)
C155—H155⋯O22^i^	0.95	2.57	3.244 (3)	128
C152—H152⋯O212^ii^	0.95	2.66	3.263 (3)	122
C153—H153⋯N21^ii^	0.95	2.74	3.682 (3)	170
C243—H243⋯O23^iii^	0.95	2.59	3.542 (4)	176
C242—H242⋯O112^iv^	0.95	2.71	3.338 (3)	124
C256—H256⋯O112^v^	0.95	2.57	3.208 (3)	125
C145—H145⋯O22^vi^	0.95	2.58	3.439 (3)	151
C153—H153⋯*Cg*5^ii^	0.95	2.61	3.469 (2)	151
C255—H255⋯*Cg*1^v^	0.95	2.66	3.544 (3)	154

Symmetry codes: (i) 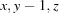; (ii) 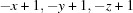; (iii) 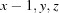; (iv) 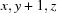; (v) 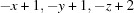; (vi) 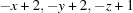.

**Table 2:** Experimental details

Crystal data	
Chemical formula	C_24_H_21_N_3_O_3_
*M*_r_	399.44
Crystal system, space group	Triclinic, *P* 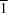
Temperature (K)	100
*a*, *b*, *c* (Å)	12.3070 (4), 13.2871 (4), 13.8499 (3)
α, β, γ (°)	90.907 (2), 100.748 (2), 109.938 (3)
*V* (Å^3^)	2083.95 (11)
*Z*	4
Radiation type	Cu *K*α
μ (mm^−1^)	0.69
Crystal size (mm)	0.52 × 0.48 × 0.24

Data collection	
Diffractometer	Agilent SuperNova, Dual, Cu at zero, Atlas
Absorption correction	Multi-scan (*CrysAlis PRO*; Agilent, 2014 ▸)
*T*_min_, *T*_max_	0.728, 1.000
No. of measured, independent and observed [*I* > 2σ(*I*)] reflections	42589, 8686, 7364
*R*_int_	0.090
(sin θ/λ)_max_ (Å^−1^)	0.630

Refinement	
*R*[*F* ^2^ > 2σ(*F* ^2^)], *wR*(*F* ^2^), *S*	0.072, 0.216, 1.09
No. of reflections	8686
No. of parameters	549
H-atom treatment	H atoms treated by a mixture of independent and constrained refinement
Δρ_max_, Δρ_min_ (e Å^−3^)	0.58, −0.39

Computer programs: *CrysAlis PRO* (Agilent, 2014 ▸), *SHELXT* (Sheldrick, 2015*a* ▸), *SHELXL2014* (Sheldrick, 2015*b* ▸) and *TITAN* (Hunter & Simpson, 1999 ▸), *Mercury* (Macrae *et al.*, 2008 ▸), *enCIFer* (Allen *et al.*, 2004 ▸), *PLATON* (Spek, 2009 ▸), *publCIF* (Westrip, 2010 ▸) and *WinGX* (Farrugia, 2012 ▸).

## supplementary crystallographic information

### Crystal data

**Table:**

C_24_H_21_N_3_O_3_	*Z* = 4
*M**_r_* = 399.44	*F*(000) = 840
Triclinic, *P*1	*D*_x_ = 1.273 Mg m^−^^3^
*a* = 12.3070 (4) Å	Cu *K*α radiation, λ = 1.54184 Å
*b* = 13.2871 (4) Å	Cell parameters from 19318 reflections
*c* = 13.8499 (3) Å	θ = 3.3–76.2°
α = 90.907 (2)°	µ = 0.69 mm^−^^1^
β = 100.748 (2)°	*T* = 100 K
γ = 109.938 (3)°	Block, yellow
*V* = 2083.95 (11) Å^3^	0.52 × 0.48 × 0.24 mm

### Data collection

**Table:**

Agilent SuperNova, Dual, Cu at zero, Atlas diffractometer	8686 independent reflections
Radiation source: SuperNova (Cu) X-ray Source	7364 reflections with *I* > 2σ(*I*)
Detector resolution: 5.1725 pixels mm^-1^	*R*_int_ = 0.090
ω scans	θ_max_ = 76.4°, θ_min_ = 3.3°
Absorption correction: multi-scan (CrysAlis PRO; Agilent, 2014)	*h* = −15→15
*T*_min_ = 0.728, *T*_max_ = 1.000	*k* = −16→16
42589 measured reflections	*l* = −17→17

### Refinement

**Table:**

Refinement on *F*^2^	0 restraints
Least-squares matrix: full	Hydrogen site location: mixed
*R*[*F*^2^ > 2σ(*F*^2^)] = 0.072	H atoms treated by a mixture of independent and constrained refinement
*wR*(*F*^2^) = 0.216	*w* = 1/[σ^2^(*F*_o_^2^) + (0.1211*P*)^2^ + 1.8979*P*] where *P* = (*F*_o_^2^ + 2*F*_c_^2^)/3
*S* = 1.09	(Δ/σ)_max_ < 0.001
8686 reflections	Δρ_max_ = 0.58 e Å^−^^3^
549 parameters	Δρ_min_ = −0.39 e Å^−^^3^

### Special details

**Table:**

Geometry. All esds (except the esd in the dihedral angle between two l.s. planes) are estimated using the full covariance matrix. The cell esds are taken into account individually in the estimation of esds in distances, angles and torsion angles; correlations between esds in cell parameters are only used when they are defined by crystal symmetry. An approximate (isotropic) treatment of cell esds is used for estimating esds involving l.s. planes.
Refinement. One low angle reflection with Fo <<< Fc that may have been affected by the beamstop was omitted from the final refinement cycles.

### Fractional atomic coordinates and isotropic or equivalent isotropic displacement parameters (Å^2^)

**Table:**

	*x*	*y*	*z*	*U*_iso_*/*U*_eq_	
N11	0.47399 (16)	0.32171 (14)	0.60492 (13)	0.0183 (4)	
C111	0.39745 (19)	0.21294 (16)	0.56313 (16)	0.0194 (4)	
H11A	0.3890	0.2091	0.4905	0.023*	
H11B	0.3181	0.1981	0.5779	0.023*	
C112	0.4456 (2)	0.12685 (17)	0.60368 (16)	0.0208 (4)	
H112	0.5289	0.1458	0.5951	0.025*	
O112	0.44400 (15)	0.12926 (13)	0.70570 (11)	0.0224 (3)	
H11O	0.451 (3)	0.071 (3)	0.731 (2)	0.034*	
C113	0.3715 (2)	0.01767 (19)	0.54911 (19)	0.0293 (5)	
H11C	0.3980	−0.0378	0.5805	0.044*	
H11D	0.3804	0.0183	0.4802	0.044*	
H11E	0.2883	0.0022	0.5514	0.044*	
C12	0.45419 (19)	0.38744 (16)	0.67156 (16)	0.0180 (4)	
C121	0.35006 (19)	0.36587 (17)	0.71663 (16)	0.0199 (4)	
C122	0.2969 (2)	0.26889 (17)	0.75549 (17)	0.0218 (4)	
H122	0.3266	0.2120	0.7520	0.026*	
C123	0.2005 (2)	0.25551 (18)	0.79922 (18)	0.0247 (5)	
H123	0.1650	0.1903	0.8269	0.030*	
C124	0.1573 (2)	0.33862 (19)	0.80183 (18)	0.0259 (5)	
N124	0.0529 (2)	0.32242 (18)	0.84456 (19)	0.0351 (5)	
O12	0.0194 (2)	0.24368 (19)	0.89045 (18)	0.0479 (6)	
O13	0.00229 (19)	0.38771 (18)	0.8305 (2)	0.0506 (6)	
C125	0.2084 (2)	0.43608 (19)	0.76456 (19)	0.0268 (5)	
H125	0.1770	0.4920	0.7670	0.032*	
C126	0.3061 (2)	0.44945 (18)	0.72384 (18)	0.0239 (5)	
H126	0.3442	0.5165	0.7003	0.029*	
N13	0.54166 (16)	0.48177 (14)	0.68955 (13)	0.0191 (4)	
C14	0.62122 (19)	0.47744 (17)	0.63342 (16)	0.0192 (4)	
C141	0.7291 (2)	0.57016 (17)	0.63058 (17)	0.0212 (4)	
C142	0.8016 (2)	0.62895 (18)	0.71690 (18)	0.0259 (5)	
H142	0.7816	0.6102	0.7788	0.031*	
C143	0.9035 (2)	0.7153 (2)	0.7129 (2)	0.0315 (5)	
H143	0.9533	0.7547	0.7721	0.038*	
C144	0.9328 (2)	0.74408 (19)	0.6222 (2)	0.0307 (5)	
H144	1.0024	0.8030	0.6196	0.037*	
C145	0.8599 (2)	0.6864 (2)	0.5359 (2)	0.0297 (5)	
H145	0.8789	0.7061	0.4738	0.036*	
C146	0.7589 (2)	0.59978 (19)	0.54048 (18)	0.0250 (5)	
H146	0.7096	0.5601	0.4812	0.030*	
C15	0.58139 (19)	0.37866 (17)	0.58023 (16)	0.0192 (4)	
C151	0.6379 (2)	0.33771 (17)	0.51106 (16)	0.0200 (4)	
C152	0.5831 (2)	0.30527 (17)	0.41259 (16)	0.0214 (4)	
H152	0.5049	0.3041	0.3895	0.026*	
C153	0.6425 (2)	0.27454 (18)	0.34782 (18)	0.0254 (5)	
H153	0.6040	0.2512	0.2811	0.030*	
C154	0.7571 (2)	0.2778 (2)	0.37998 (19)	0.0291 (5)	
H154	0.7975	0.2575	0.3354	0.035*	
C155	0.8127 (2)	0.3108 (2)	0.4775 (2)	0.0308 (5)	
H155	0.8915	0.3134	0.4998	0.037*	
C156	0.7531 (2)	0.34025 (19)	0.54298 (17)	0.0254 (5)	
H156	0.7915	0.3622	0.6100	0.030*	
N21	0.53514 (16)	0.84449 (14)	0.89788 (13)	0.0178 (4)	
C211	0.6129 (2)	0.78650 (17)	0.94029 (16)	0.0211 (4)	
H21A	0.6196	0.7890	1.0127	0.025*	
H21B	0.6928	0.8231	0.9271	0.025*	
C212	0.5678 (2)	0.66925 (18)	0.89808 (16)	0.0218 (4)	
H212	0.4833	0.6350	0.9034	0.026*	
O212	0.57451 (15)	0.67128 (13)	0.79728 (12)	0.0247 (4)	
H21O	0.559 (3)	0.606 (3)	0.769 (3)	0.037*	
C213	0.6397 (3)	0.6084 (2)	0.9554 (2)	0.0331 (6)	
H21C	0.6128	0.5346	0.9252	0.050*	
H21D	0.6294	0.6073	1.0239	0.050*	
H21E	0.7234	0.6442	0.9540	0.050*	
C22	0.55613 (19)	0.92431 (16)	0.83472 (16)	0.0186 (4)	
C221	0.65812 (19)	0.96701 (17)	0.78737 (16)	0.0193 (4)	
C222	0.6966 (2)	1.07800 (18)	0.77616 (17)	0.0220 (4)	
H222	0.6612	1.1216	0.8040	0.026*	
C223	0.7853 (2)	1.12438 (19)	0.72527 (18)	0.0242 (5)	
H223	0.8119	1.1994	0.7184	0.029*	
C224	0.8346 (2)	1.05887 (19)	0.68452 (17)	0.0236 (5)	
N224	0.92519 (18)	1.10743 (18)	0.62720 (16)	0.0287 (4)	
O22	0.96751 (17)	1.20609 (16)	0.63188 (15)	0.0366 (4)	
O23	0.9549 (2)	1.04823 (19)	0.57759 (18)	0.0479 (6)	
C225	0.7994 (2)	0.94926 (19)	0.69411 (17)	0.0235 (5)	
H225	0.8348	0.9063	0.6653	0.028*	
C226	0.7115 (2)	0.90354 (18)	0.74671 (17)	0.0224 (4)	
H226	0.6874	0.8288	0.7551	0.027*	
N23	0.46808 (16)	0.96207 (14)	0.81808 (14)	0.0192 (4)	
C24	0.38696 (19)	0.90515 (17)	0.87159 (16)	0.0198 (4)	
C241	0.27705 (19)	0.92635 (17)	0.87218 (17)	0.0203 (4)	
C242	0.2189 (2)	0.95697 (18)	0.78775 (17)	0.0225 (4)	
H242	0.2500	0.9640	0.7294	0.027*	
C243	0.1155 (2)	0.97731 (18)	0.7888 (2)	0.0265 (5)	
H243	0.0764	0.9980	0.7309	0.032*	
C244	0.0689 (2)	0.96790 (19)	0.8728 (2)	0.0299 (5)	
H244	−0.0018	0.9819	0.8728	0.036*	
C245	0.1262 (2)	0.9377 (2)	0.9576 (2)	0.0298 (5)	
H245	0.0946	0.9310	1.0157	0.036*	
C246	0.2299 (2)	0.91744 (19)	0.95732 (18)	0.0258 (5)	
H246	0.2690	0.8974	1.0155	0.031*	
C25	0.42676 (19)	0.83105 (17)	0.92131 (16)	0.0188 (4)	
C251	0.3663 (2)	0.74902 (17)	0.98272 (16)	0.0197 (4)	
C252	0.2548 (2)	0.67459 (19)	0.94172 (18)	0.0255 (5)	
H252	0.2227	0.6737	0.8737	0.031*	
C253	0.1905 (2)	0.6023 (2)	0.9986 (2)	0.0310 (5)	
H253	0.1147	0.5522	0.9697	0.037*	
C254	0.2371 (2)	0.6029 (2)	1.0986 (2)	0.0306 (5)	
H254	0.1930	0.5537	1.1381	0.037*	
C255	0.3483 (2)	0.6756 (2)	1.13974 (18)	0.0271 (5)	
H255	0.3807	0.6755	1.2076	0.032*	
C256	0.4129 (2)	0.74873 (18)	1.08274 (17)	0.0226 (4)	
H256	0.4888	0.7986	1.1118	0.027*	

### Atomic displacement parameters (Å^2^)

**Table:**

	*U*^11^	*U*^22^	*U*^33^	*U*^12^	*U*^13^	*U*^23^
N11	0.0253 (9)	0.0140 (8)	0.0201 (9)	0.0118 (7)	0.0059 (7)	0.0034 (7)
C111	0.0244 (10)	0.0148 (9)	0.0207 (10)	0.0093 (8)	0.0038 (8)	0.0015 (8)
C112	0.0305 (11)	0.0165 (10)	0.0200 (10)	0.0135 (8)	0.0061 (8)	0.0021 (8)
O112	0.0357 (9)	0.0183 (7)	0.0199 (8)	0.0173 (7)	0.0061 (6)	0.0033 (6)
C113	0.0416 (13)	0.0169 (11)	0.0304 (12)	0.0128 (10)	0.0050 (10)	−0.0001 (9)
C12	0.0248 (10)	0.0135 (9)	0.0193 (10)	0.0109 (8)	0.0052 (8)	0.0018 (7)
C121	0.0233 (10)	0.0173 (10)	0.0218 (10)	0.0106 (8)	0.0048 (8)	0.0005 (8)
C122	0.0272 (11)	0.0151 (10)	0.0270 (11)	0.0113 (8)	0.0080 (9)	0.0017 (8)
C123	0.0284 (11)	0.0194 (10)	0.0277 (11)	0.0082 (9)	0.0091 (9)	0.0015 (9)
C124	0.0248 (11)	0.0242 (11)	0.0312 (12)	0.0099 (9)	0.0101 (9)	−0.0026 (9)
N124	0.0310 (11)	0.0307 (11)	0.0463 (13)	0.0090 (9)	0.0183 (10)	−0.0043 (10)
O12	0.0452 (12)	0.0458 (12)	0.0606 (14)	0.0141 (10)	0.0324 (11)	0.0132 (10)
O13	0.0404 (11)	0.0397 (12)	0.0850 (18)	0.0215 (9)	0.0314 (11)	0.0010 (11)
C125	0.0303 (12)	0.0203 (11)	0.0356 (13)	0.0145 (9)	0.0104 (10)	−0.0013 (9)
C126	0.0305 (11)	0.0164 (10)	0.0288 (11)	0.0118 (9)	0.0088 (9)	0.0025 (8)
N13	0.0259 (9)	0.0143 (8)	0.0212 (9)	0.0107 (7)	0.0080 (7)	0.0025 (7)
C14	0.0257 (10)	0.0165 (10)	0.0198 (10)	0.0123 (8)	0.0060 (8)	0.0031 (8)
C141	0.0259 (10)	0.0155 (10)	0.0277 (11)	0.0123 (8)	0.0087 (9)	0.0036 (8)
C142	0.0323 (12)	0.0196 (11)	0.0281 (12)	0.0108 (9)	0.0089 (9)	−0.0009 (9)
C143	0.0321 (12)	0.0222 (11)	0.0398 (14)	0.0090 (10)	0.0080 (11)	−0.0041 (10)
C144	0.0280 (12)	0.0204 (11)	0.0485 (15)	0.0096 (9)	0.0172 (11)	0.0038 (10)
C145	0.0340 (12)	0.0254 (12)	0.0382 (13)	0.0150 (10)	0.0187 (11)	0.0098 (10)
C146	0.0308 (11)	0.0220 (11)	0.0270 (11)	0.0129 (9)	0.0098 (9)	0.0046 (9)
C15	0.0249 (10)	0.0163 (10)	0.0203 (10)	0.0119 (8)	0.0051 (8)	0.0043 (8)
C151	0.0283 (11)	0.0144 (9)	0.0234 (10)	0.0133 (8)	0.0088 (9)	0.0048 (8)
C152	0.0275 (11)	0.0167 (10)	0.0229 (11)	0.0110 (8)	0.0059 (9)	0.0028 (8)
C153	0.0375 (12)	0.0181 (10)	0.0243 (11)	0.0133 (9)	0.0087 (9)	−0.0004 (8)
C154	0.0421 (14)	0.0264 (12)	0.0300 (12)	0.0218 (10)	0.0156 (10)	0.0025 (9)
C155	0.0335 (12)	0.0344 (13)	0.0355 (13)	0.0240 (11)	0.0101 (10)	0.0058 (10)
C156	0.0314 (12)	0.0273 (11)	0.0237 (11)	0.0186 (9)	0.0049 (9)	0.0040 (9)
N21	0.0241 (9)	0.0145 (8)	0.0189 (8)	0.0117 (7)	0.0048 (7)	0.0007 (7)
C211	0.0283 (11)	0.0187 (10)	0.0216 (10)	0.0163 (9)	0.0027 (8)	0.0011 (8)
C212	0.0310 (11)	0.0184 (10)	0.0215 (10)	0.0146 (9)	0.0067 (9)	0.0025 (8)
O212	0.0402 (9)	0.0160 (7)	0.0231 (8)	0.0148 (7)	0.0092 (7)	0.0009 (6)
C213	0.0511 (15)	0.0275 (12)	0.0315 (13)	0.0284 (12)	0.0065 (11)	0.0046 (10)
C22	0.0260 (10)	0.0125 (9)	0.0204 (10)	0.0108 (8)	0.0049 (8)	−0.0001 (7)
C221	0.0218 (10)	0.0197 (10)	0.0199 (10)	0.0122 (8)	0.0032 (8)	0.0010 (8)
C222	0.0260 (11)	0.0201 (10)	0.0241 (11)	0.0132 (8)	0.0055 (8)	0.0019 (8)
C223	0.0265 (11)	0.0207 (10)	0.0280 (11)	0.0115 (9)	0.0054 (9)	0.0055 (9)
C224	0.0228 (10)	0.0279 (12)	0.0240 (11)	0.0137 (9)	0.0048 (8)	0.0060 (9)
N224	0.0281 (10)	0.0347 (11)	0.0289 (10)	0.0159 (9)	0.0093 (8)	0.0099 (9)
O22	0.0342 (9)	0.0351 (10)	0.0410 (11)	0.0093 (8)	0.0134 (8)	0.0129 (8)
O23	0.0586 (13)	0.0499 (13)	0.0568 (14)	0.0314 (11)	0.0390 (11)	0.0159 (11)
C225	0.0264 (11)	0.0251 (11)	0.0249 (11)	0.0160 (9)	0.0064 (9)	0.0028 (9)
C226	0.0278 (11)	0.0190 (10)	0.0250 (11)	0.0141 (9)	0.0055 (9)	0.0022 (8)
N23	0.0245 (9)	0.0161 (8)	0.0221 (9)	0.0129 (7)	0.0061 (7)	0.0017 (7)
C24	0.0257 (10)	0.0181 (10)	0.0197 (10)	0.0125 (8)	0.0055 (8)	0.0001 (8)
C241	0.0248 (10)	0.0141 (9)	0.0260 (11)	0.0112 (8)	0.0070 (8)	−0.0004 (8)
C242	0.0240 (10)	0.0191 (10)	0.0275 (11)	0.0107 (8)	0.0065 (9)	0.0033 (8)
C243	0.0244 (11)	0.0196 (10)	0.0388 (13)	0.0126 (9)	0.0046 (9)	0.0032 (9)
C244	0.0261 (11)	0.0218 (11)	0.0464 (15)	0.0127 (9)	0.0110 (10)	−0.0016 (10)
C245	0.0348 (13)	0.0279 (12)	0.0353 (13)	0.0172 (10)	0.0164 (10)	−0.0003 (10)
C246	0.0339 (12)	0.0241 (11)	0.0266 (11)	0.0175 (9)	0.0098 (9)	0.0015 (9)
C25	0.0253 (10)	0.0162 (9)	0.0188 (10)	0.0123 (8)	0.0041 (8)	−0.0021 (8)
C251	0.0286 (11)	0.0165 (10)	0.0204 (10)	0.0144 (8)	0.0082 (8)	0.0019 (8)
C252	0.0301 (11)	0.0233 (11)	0.0257 (11)	0.0128 (9)	0.0057 (9)	0.0001 (9)
C253	0.0321 (12)	0.0240 (12)	0.0392 (14)	0.0112 (10)	0.0104 (10)	0.0030 (10)
C254	0.0412 (13)	0.0238 (11)	0.0394 (14)	0.0201 (10)	0.0211 (11)	0.0133 (10)
C255	0.0420 (13)	0.0282 (12)	0.0244 (11)	0.0255 (10)	0.0136 (10)	0.0095 (9)
C256	0.0319 (11)	0.0205 (10)	0.0225 (11)	0.0176 (9)	0.0066 (9)	0.0018 (8)

### Geometric parameters (Å, º)

**Table:**

N11—C12	1.370 (3)	N21—C22	1.372 (3)
N11—C15	1.390 (3)	N21—C25	1.385 (3)
N11—C111	1.467 (3)	N21—C211	1.471 (3)
C111—C112	1.527 (3)	C211—C212	1.529 (3)
C111—H11A	0.9900	C211—H21A	0.9900
C111—H11B	0.9900	C211—H21B	0.9900
C112—O112	1.417 (3)	C212—O212	1.414 (3)
C112—C113	1.521 (3)	C212—C213	1.521 (3)
C112—H112	1.0000	C212—H212	1.0000
O112—H11O	0.88 (4)	O212—H21O	0.89 (4)
C113—H11C	0.9800	C213—H21C	0.9800
C113—H11D	0.9800	C213—H21D	0.9800
C113—H11E	0.9800	C213—H21E	0.9800
C12—N13	1.327 (3)	C22—N23	1.327 (3)
C12—C121	1.472 (3)	C22—C221	1.470 (3)
C121—C122	1.399 (3)	C221—C226	1.400 (3)
C121—C126	1.401 (3)	C221—C222	1.408 (3)
C122—C123	1.389 (3)	C222—C223	1.382 (3)
C122—H122	0.9500	C222—H222	0.9500
C123—C124	1.382 (3)	C223—C224	1.384 (3)
C123—H123	0.9500	C223—H223	0.9500
C124—C125	1.388 (3)	C224—C225	1.387 (3)
C124—N124	1.465 (3)	C224—N224	1.465 (3)
N124—O12	1.222 (3)	N224—O23	1.224 (3)
N124—O13	1.228 (3)	N224—O22	1.230 (3)
C125—C126	1.381 (3)	C225—C226	1.390 (3)
C125—H125	0.9500	C225—H225	0.9500
C126—H126	0.9500	C226—H226	0.9500
N13—C14	1.374 (3)	N23—C24	1.377 (3)
C14—C15	1.379 (3)	C24—C25	1.381 (3)
C14—C141	1.479 (3)	C24—C241	1.474 (3)
C141—C142	1.391 (3)	C241—C242	1.395 (3)
C141—C146	1.391 (3)	C241—C246	1.398 (3)
C142—C143	1.392 (3)	C242—C243	1.391 (3)
C142—H142	0.9500	C242—H242	0.9500
C143—C144	1.394 (4)	C243—C244	1.380 (4)
C143—H143	0.9500	C243—H243	0.9500
C144—C145	1.387 (4)	C244—C245	1.391 (4)
C144—H144	0.9500	C244—H244	0.9500
C145—C146	1.389 (3)	C245—C246	1.392 (3)
C145—H145	0.9500	C245—H245	0.9500
C146—H146	0.9500	C246—H246	0.9500
C15—C151	1.479 (3)	C25—C251	1.475 (3)
C151—C152	1.393 (3)	C251—C252	1.397 (3)
C151—C156	1.394 (3)	C251—C256	1.398 (3)
C152—C153	1.394 (3)	C252—C253	1.381 (4)
C152—H152	0.9500	C252—H252	0.9500
C153—C154	1.383 (4)	C253—C254	1.395 (4)
C153—H153	0.9500	C253—H253	0.9500
C154—C155	1.384 (4)	C254—C255	1.386 (4)
C154—H154	0.9500	C254—H254	0.9500
C155—C156	1.395 (3)	C255—C256	1.390 (3)
C155—H155	0.9500	C255—H255	0.9500
C156—H156	0.9500	C256—H256	0.9500
			
C12—N11—C15	107.00 (18)	C22—N21—C25	107.17 (17)
C12—N11—C111	128.14 (18)	C22—N21—C211	128.44 (18)
C15—N11—C111	124.83 (18)	C25—N21—C211	124.28 (18)
N11—C111—C112	112.57 (17)	N21—C211—C212	112.49 (18)
N11—C111—H11A	109.1	N21—C211—H21A	109.1
C112—C111—H11A	109.1	C212—C211—H21A	109.1
N11—C111—H11B	109.1	N21—C211—H21B	109.1
C112—C111—H11B	109.1	C212—C211—H21B	109.1
H11A—C111—H11B	107.8	H21A—C211—H21B	107.8
O112—C112—C113	112.54 (18)	O212—C212—C213	113.03 (19)
O112—C112—C111	106.18 (17)	O212—C212—C211	106.32 (17)
C113—C112—C111	110.42 (18)	C213—C212—C211	110.40 (19)
O112—C112—H112	109.2	O212—C212—H212	109.0
C113—C112—H112	109.2	C213—C212—H212	109.0
C111—C112—H112	109.2	C211—C212—H212	109.0
C112—O112—H11O	111 (2)	C212—O212—H21O	112 (2)
C112—C113—H11C	109.5	C212—C213—H21C	109.5
C112—C113—H11D	109.5	C212—C213—H21D	109.5
H11C—C113—H11D	109.5	H21C—C213—H21D	109.5
C112—C113—H11E	109.5	C212—C213—H21E	109.5
H11C—C113—H11E	109.5	H21C—C213—H21E	109.5
H11D—C113—H11E	109.5	H21D—C213—H21E	109.5
N13—C12—N11	110.95 (19)	N23—C22—N21	110.80 (19)
N13—C12—C121	121.03 (19)	N23—C22—C221	120.87 (19)
N11—C12—C121	127.94 (19)	N21—C22—C221	128.32 (18)
C122—C121—C126	119.1 (2)	C226—C221—C222	119.1 (2)
C122—C121—C12	123.53 (19)	C226—C221—C22	124.2 (2)
C126—C121—C12	117.30 (19)	C222—C221—C22	116.45 (19)
C123—C122—C121	120.2 (2)	C223—C222—C221	120.9 (2)
C123—C122—H122	119.9	C223—C222—H222	119.5
C121—C122—H122	119.9	C221—C222—H222	119.5
C124—C123—C122	118.9 (2)	C222—C223—C224	118.4 (2)
C124—C123—H123	120.6	C222—C223—H223	120.8
C122—C123—H123	120.6	C224—C223—H223	120.8
C123—C124—C125	122.4 (2)	C223—C224—C225	122.5 (2)
C123—C124—N124	118.6 (2)	C223—C224—N224	118.3 (2)
C125—C124—N124	119.0 (2)	C225—C224—N224	119.2 (2)
O12—N124—O13	123.5 (2)	O23—N224—O22	123.5 (2)
O12—N124—C124	118.4 (2)	O23—N224—C224	118.6 (2)
O13—N124—C124	118.1 (2)	O22—N224—C224	117.8 (2)
C126—C125—C124	118.1 (2)	C224—C225—C226	118.7 (2)
C126—C125—H125	120.9	C224—C225—H225	120.6
C124—C125—H125	120.9	C226—C225—H225	120.6
C125—C126—C121	121.2 (2)	C225—C226—C221	120.3 (2)
C125—C126—H126	119.4	C225—C226—H226	119.8
C121—C126—H126	119.4	C221—C226—H226	119.8
C12—N13—C14	106.47 (18)	C22—N23—C24	106.57 (18)
N13—C14—C15	109.86 (19)	N23—C24—C25	109.58 (19)
N13—C14—C141	122.40 (19)	N23—C24—C241	122.00 (19)
C15—C14—C141	127.7 (2)	C25—C24—C241	128.4 (2)
C142—C141—C146	119.1 (2)	C242—C241—C246	118.8 (2)
C142—C141—C14	121.0 (2)	C242—C241—C24	120.4 (2)
C146—C141—C14	119.9 (2)	C246—C241—C24	120.8 (2)
C141—C142—C143	120.2 (2)	C243—C242—C241	120.2 (2)
C141—C142—H142	119.9	C243—C242—H242	119.9
C143—C142—H142	119.9	C241—C242—H242	119.9
C142—C143—C144	120.2 (2)	C244—C243—C242	120.9 (2)
C142—C143—H143	119.9	C244—C243—H243	119.6
C144—C143—H143	119.9	C242—C243—H243	119.6
C145—C144—C143	119.7 (2)	C243—C244—C245	119.5 (2)
C145—C144—H144	120.1	C243—C244—H244	120.3
C143—C144—H144	120.1	C245—C244—H244	120.3
C144—C145—C146	119.8 (2)	C244—C245—C246	120.1 (2)
C144—C145—H145	120.1	C244—C245—H245	120.0
C146—C145—H145	120.1	C246—C245—H245	120.0
C145—C146—C141	121.0 (2)	C245—C246—C241	120.6 (2)
C145—C146—H146	119.5	C245—C246—H246	119.7
C141—C146—H146	119.5	C241—C246—H246	119.7
C14—C15—N11	105.72 (19)	C24—C25—N21	105.88 (19)
C14—C15—C151	128.9 (2)	C24—C25—C251	128.6 (2)
N11—C15—C151	125.36 (19)	N21—C25—C251	125.49 (18)
C152—C151—C156	118.9 (2)	C252—C251—C256	118.8 (2)
C152—C151—C15	121.9 (2)	C252—C251—C25	118.9 (2)
C156—C151—C15	119.0 (2)	C256—C251—C25	122.0 (2)
C151—C152—C153	120.2 (2)	C253—C252—C251	121.0 (2)
C151—C152—H152	119.9	C253—C252—H252	119.5
C153—C152—H152	119.9	C251—C252—H252	119.5
C154—C153—C152	120.5 (2)	C252—C253—C254	119.9 (2)
C154—C153—H153	119.8	C252—C253—H253	120.1
C152—C153—H153	119.7	C254—C253—H253	120.1
C153—C154—C155	119.7 (2)	C255—C254—C253	119.6 (2)
C153—C154—H154	120.2	C255—C254—H254	120.2
C155—C154—H154	120.2	C253—C254—H254	120.2
C154—C155—C156	120.1 (2)	C254—C255—C256	120.6 (2)
C154—C155—H155	119.9	C254—C255—H255	119.7
C156—C155—H155	119.9	C256—C255—H255	119.7
C151—C156—C155	120.6 (2)	C255—C256—C251	120.1 (2)
C151—C156—H156	119.7	C255—C256—H256	120.0
C155—C156—H156	119.7	C251—C256—H256	120.0
			
C12—N11—C111—C112	−107.9 (2)	C22—N21—C211—C212	−110.1 (2)
C15—N11—C111—C112	74.2 (2)	C25—N21—C211—C212	74.3 (3)
N11—C111—C112—O112	64.7 (2)	N21—C211—C212—O212	66.0 (2)
N11—C111—C112—C113	−173.05 (19)	N21—C211—C212—C213	−171.0 (2)
C15—N11—C12—N13	0.4 (2)	C25—N21—C22—N23	0.6 (2)
C111—N11—C12—N13	−177.80 (18)	C211—N21—C22—N23	−175.67 (19)
C15—N11—C12—C121	177.1 (2)	C25—N21—C22—C221	−178.8 (2)
C111—N11—C12—C121	−1.1 (3)	C211—N21—C22—C221	4.9 (3)
N13—C12—C121—C122	−138.1 (2)	N23—C22—C221—C226	−137.8 (2)
N11—C12—C121—C122	45.5 (3)	N21—C22—C221—C226	41.5 (3)
N13—C12—C121—C126	39.2 (3)	N23—C22—C221—C222	36.9 (3)
N11—C12—C121—C126	−137.1 (2)	N21—C22—C221—C222	−143.8 (2)
C126—C121—C122—C123	1.0 (3)	C226—C221—C222—C223	0.4 (3)
C12—C121—C122—C123	178.3 (2)	C22—C221—C222—C223	−174.5 (2)
C121—C122—C123—C124	1.2 (4)	C221—C222—C223—C224	0.7 (3)
C122—C123—C124—C125	−1.7 (4)	C222—C223—C224—C225	−0.9 (4)
C122—C123—C124—N124	177.6 (2)	C222—C223—C224—N224	177.6 (2)
C123—C124—N124—O12	11.7 (4)	C223—C224—N224—O23	−168.0 (2)
C125—C124—N124—O12	−169.0 (3)	C225—C224—N224—O23	10.5 (3)
C123—C124—N124—O13	−166.8 (3)	C223—C224—N224—O22	12.1 (3)
C125—C124—N124—O13	12.5 (4)	C225—C224—N224—O22	−169.3 (2)
C123—C124—C125—C126	−0.2 (4)	C223—C224—C225—C226	0.0 (4)
N124—C124—C125—C126	−179.5 (2)	N224—C224—C225—C226	−178.5 (2)
C124—C125—C126—C121	2.4 (4)	C224—C225—C226—C221	1.2 (3)
C122—C121—C126—C125	−2.9 (4)	C222—C221—C226—C225	−1.4 (3)
C12—C121—C126—C125	179.6 (2)	C22—C221—C226—C225	173.1 (2)
N11—C12—N13—C14	−0.3 (2)	N21—C22—N23—C24	−0.2 (2)
C121—C12—N13—C14	−177.24 (19)	C221—C22—N23—C24	179.24 (19)
C12—N13—C14—C15	0.0 (2)	C22—N23—C24—C25	−0.3 (2)
C12—N13—C14—C141	177.4 (2)	C22—N23—C24—C241	179.2 (2)
N13—C14—C141—C142	46.6 (3)	N23—C24—C241—C242	34.5 (3)
C15—C14—C141—C142	−136.5 (2)	C25—C24—C241—C242	−146.2 (2)
N13—C14—C141—C146	−133.2 (2)	N23—C24—C241—C246	−144.8 (2)
C15—C14—C141—C146	43.6 (3)	C25—C24—C241—C246	34.5 (3)
C146—C141—C142—C143	−0.9 (3)	C246—C241—C242—C243	−0.5 (3)
C14—C141—C142—C143	179.3 (2)	C24—C241—C242—C243	−179.7 (2)
C141—C142—C143—C144	0.7 (4)	C241—C242—C243—C244	0.1 (3)
C142—C143—C144—C145	0.1 (4)	C242—C243—C244—C245	0.0 (4)
C143—C144—C145—C146	−0.7 (4)	C243—C244—C245—C246	0.1 (4)
C144—C145—C146—C141	0.5 (4)	C244—C245—C246—C241	−0.4 (4)
C142—C141—C146—C145	0.3 (3)	C242—C241—C246—C245	0.6 (3)
C14—C141—C146—C145	−179.9 (2)	C24—C241—C246—C245	179.9 (2)
N13—C14—C15—N11	0.2 (2)	N23—C24—C25—N21	0.6 (2)
C141—C14—C15—N11	−177.0 (2)	C241—C24—C25—N21	−178.8 (2)
N13—C14—C15—C151	−179.5 (2)	N23—C24—C25—C251	−176.2 (2)
C141—C14—C15—C151	3.4 (4)	C241—C24—C25—C251	4.4 (4)
C12—N11—C15—C14	−0.4 (2)	C22—N21—C25—C24	−0.7 (2)
C111—N11—C15—C14	177.92 (18)	C211—N21—C25—C24	175.74 (19)
C12—N11—C15—C151	179.31 (19)	C22—N21—C25—C251	176.2 (2)
C111—N11—C15—C151	−2.4 (3)	C211—N21—C25—C251	−7.3 (3)
C14—C15—C151—C152	−120.2 (3)	C24—C25—C251—C252	56.1 (3)
N11—C15—C151—C152	60.2 (3)	N21—C25—C251—C252	−120.1 (2)
C14—C15—C151—C156	54.2 (3)	C24—C25—C251—C256	−118.7 (3)
N11—C15—C151—C156	−125.4 (2)	N21—C25—C251—C256	65.1 (3)
C156—C151—C152—C153	0.9 (3)	C256—C251—C252—C253	0.6 (3)
C15—C151—C152—C153	175.3 (2)	C25—C251—C252—C253	−174.4 (2)
C151—C152—C153—C154	−1.2 (3)	C251—C252—C253—C254	−0.1 (4)
C152—C153—C154—C155	0.7 (4)	C252—C253—C254—C255	−0.6 (4)
C153—C154—C155—C156	0.2 (4)	C253—C254—C255—C256	0.9 (3)
C152—C151—C156—C155	0.0 (3)	C254—C255—C256—C251	−0.4 (3)
C15—C151—C156—C155	−174.6 (2)	C252—C251—C256—C255	−0.3 (3)
C154—C155—C156—C151	−0.5 (4)	C25—C251—C256—C255	174.50 (19)

### Hydrogen-bond geometry (Å, º)

Cg1 and Cg5 are the centroids of the N11/C12/N13/C14/C15 and N21/C22/N23/C24/C25 rings, respectively.

**Table:**

*D*—H···*A*	*D*—H	H···*A*	*D*···*A*	*D*—H···*A*
O212—H21*O*···N13	0.89 (4)	1.90 (4)	2.773 (2)	168 (3)
C253—H253···O13	0.95	2.69	3.491 (4)	143
O112—H11*O*···N23^i^	0.88 (4)	1.94 (4)	2.798 (2)	165 (3)
C155—H155···O22^i^	0.95	2.57	3.244 (3)	128
C152—H152···O212^ii^	0.95	2.66	3.263 (3)	122
C153—H153···N21^ii^	0.95	2.74	3.682 (3)	170
C243—H243···O23^iii^	0.95	2.59	3.542 (4)	176
C242—H242···O112^iv^	0.95	2.71	3.338 (3)	124
C256—H256···O112^v^	0.95	2.57	3.208 (3)	125
C145—H145···O22^vi^	0.95	2.58	3.439 (3)	151
C153—H153···*Cg*5^ii^	0.95	2.61	3.469 (2)	151
C255—H255···*Cg*1^v^	0.95	2.66	3.544 (3)	154

Symmetry codes: (i) *x*, *y*−1, *z*; (ii) −*x*+1, −*y*+1, −*z*+1; (iii) *x*−1, *y*, *z*; (iv) *x*, *y*+1, *z*; (v) −*x*+1, −*y*+1, −*z*+2; (vi) −*x*+2, −*y*+2, −*z*+1.
